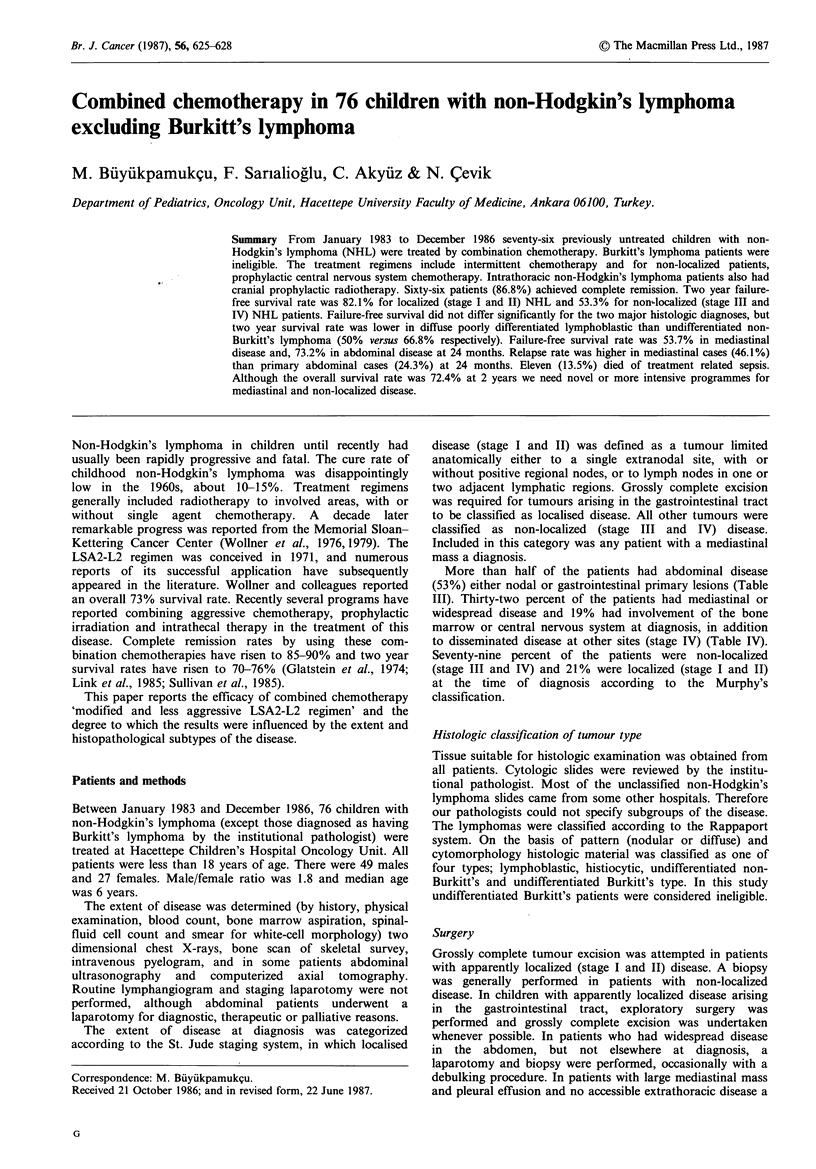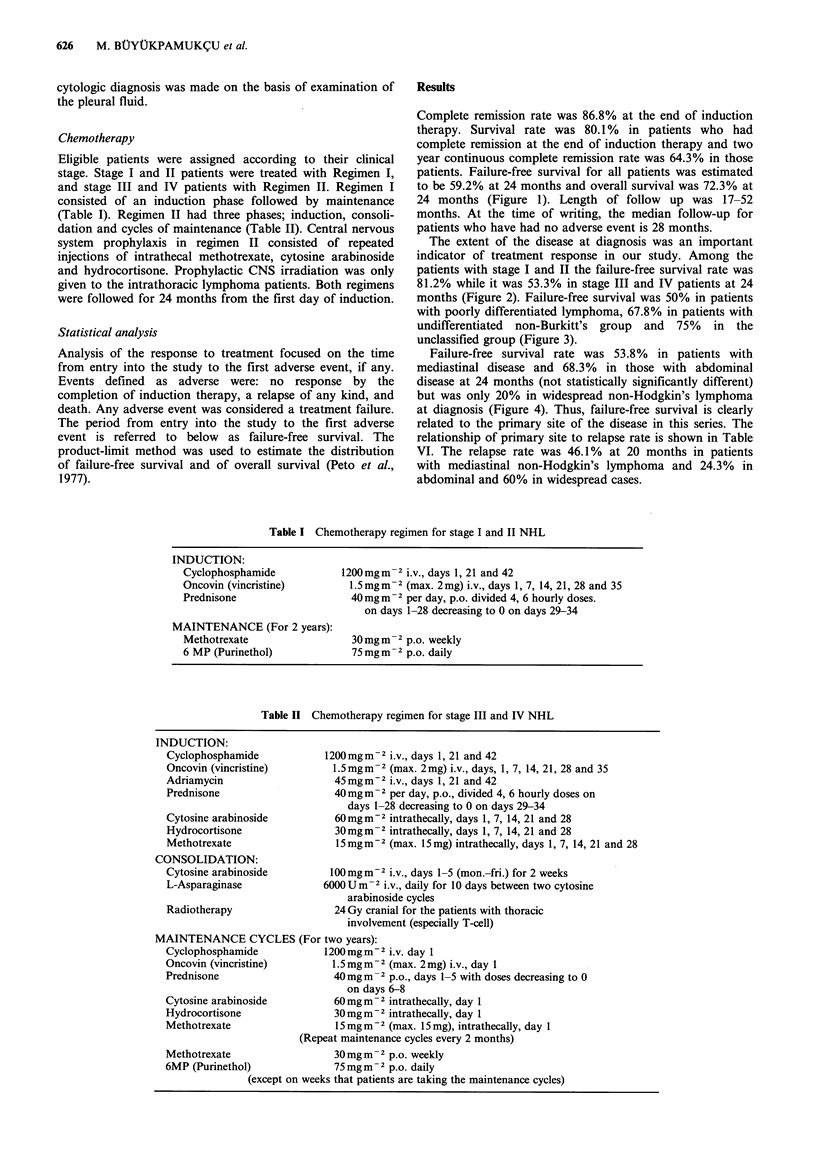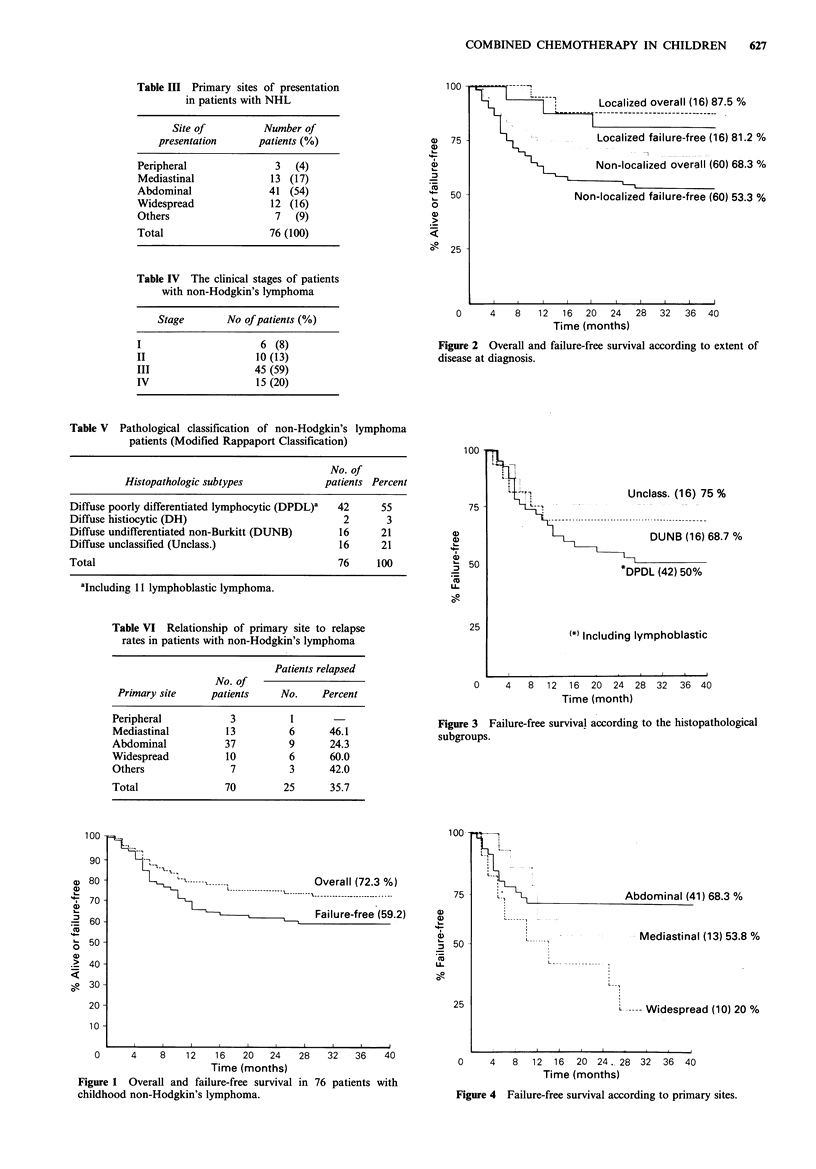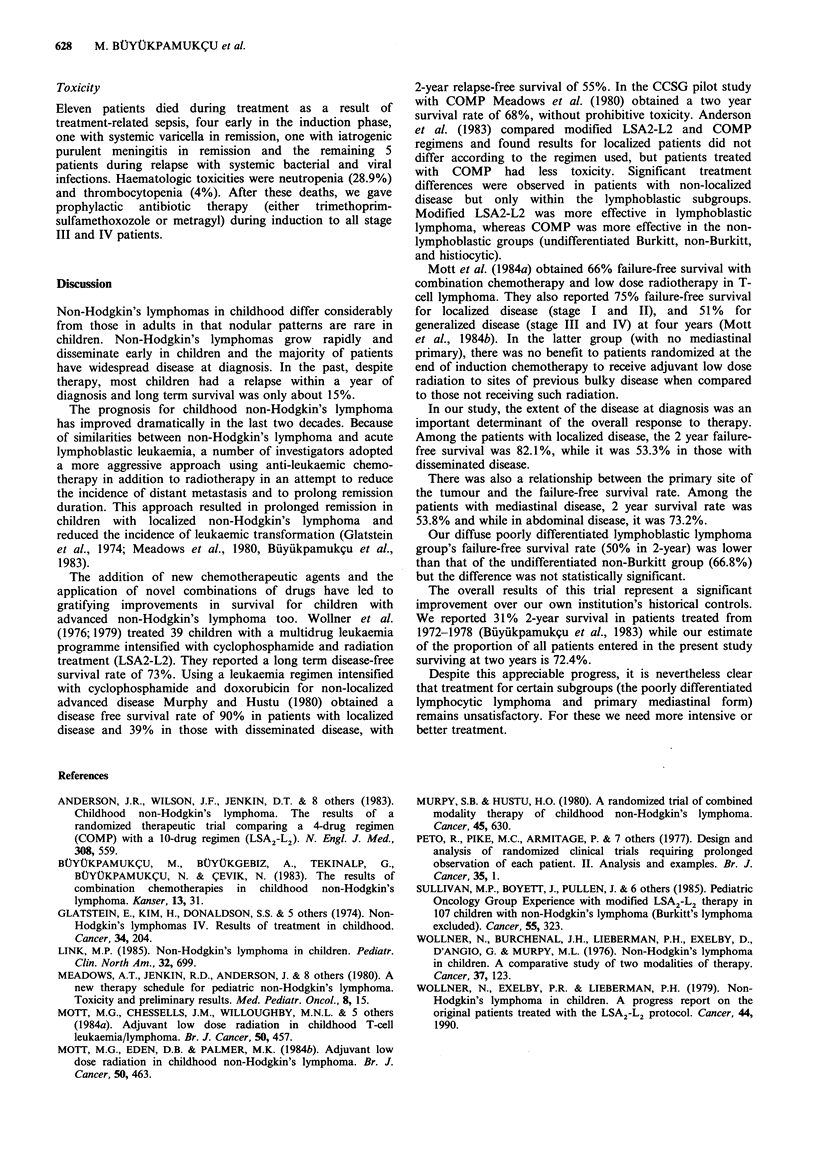# Combined chemotherapy in 76 children with non-Hodgkin's lymphoma excluding Burkitt's lymphoma.

**DOI:** 10.1038/bjc.1987.255

**Published:** 1987-11

**Authors:** M. Büyükpamukçu, F. Sarialioğlu, C. Akyüz, N. Cevik

**Affiliations:** Department of Pediatrics, Hacettepe University, Faculty of Medicine, Ankara, Turkey.

## Abstract

From January 1983 to December 1986 seventy-six previously untreated children with non-Hodgkin's lymphoma (NHL) were treated by combination chemotherapy. Burkitt's lymphoma patients were ineligible. The treatment regimens include intermittent chemotherapy and for non-localized patients, prophylactic central nervous system chemotherapy. Intrathoracic non-Hodgkin's lymphoma patients also had cranial prophylactic radiotherapy. Sixty-six patients (86.8%) achieved complete remission. Two year failure-free survival rate was 82.1% for localized (stage I and II) NHL and 53.3% for non-localized (stage III and IV) NHL patients. Failure-free survival did not differ significantly for the two major histologic diagnoses, but two year survival rate was lower in diffuse poorly differentiated lymphoblastic than undifferentiated non-Burkitt's lymphoma (50% versus 66.8% respectively). Failure-free survival rate was 53.7% in mediastinal disease and, 73.2% in abdominal disease at 24 months. Relapse rate was higher in mediastinal cases (46.1%) than primary abdominal cases (24.3%) at 24 months. Eleven (13.5%) died of treatment related sepsis. Although the overall survival rate was 72.4% at 2 years we need novel or more intensive programmes for mediastinal and non-localized disease.


*Br. J. Cancer* (1987), **56**, 625–628 © The Macmillan Press Ltd., 1987

### Combined chemotherapy in 76 children with non-Hodgkin's lymphoma excluding Burkitt's lymphoma

M. Büyükpamukçu, F. Sarıalioğlu, C. Akyüz & N. Çevik

*Department of Pediatrics, Oncology Unit, Hacettepe University Faculty of Medicine, Ankara 06100, Turkey.*

**Summary** From January 1983 to December 1986 seventy-six previously untreated children with non-Hodgkin's lymphoma (NHL) were treated by combination chemotherapy. Burkitt's lymphoma patients were ineligible. The treatment regimens include intermittent chemotherapy and for non-localized patients, prophylactic central nervous system chemotherapy. Intrathoracic non-Hodgkin's lymphoma patients also had cranial prophylactic radiotherapy. Sixty-six patients (86.8%) achieved complete remission. Two year failure-free survival rate was 82.1% for localized (stage I and II) NHL and 53.3% for non-localized (stage III and IV) NHL patients. Failure-free survival did not differ significantly for the two major histologic diagnoses, but two year survival rate was lower in diffuse poorly differentiated lymphoblastic than undifferentiated non-Burkitt's lymphoma (50% *versus* 66.8% respectively). Failure-free survival rate was 53.7% in mediastinal disease and, 73.2% in abdominal disease at 24 months. Relapse rate was higher in mediastinal cases (46.1%) than primary abdominal cases (24.3%) at 24 months. Eleven (13.5%) died of treatment related sepsis. Although the overall survival rate was 72.4% at 2 years we need novel or more intensive programmes for mediastinal and non-localized disease.

Non-Hodgkin's lymphoma in children until recently had usually been rapidly progressive and fatal. The cure rate of childhood non-Hodgkin's lymphoma was disappointingly low in the 1960s, about 10–15%. Treatment regimens generally included radiotherapy to involved areas, with or without single agent chemotherapy. A decade later remarkable progress was reported from the Memorial Sloan–Kettering Cancer Center (Wollner *et al.*, 1976, 1979). The LSA2-L2 regimen was conceived in 1971, and numerous reports of its successful application have subsequently appeared in the literature. Wollner and colleagues reported an overall 73% survival rate. Recently several programs have reported combining aggressive chemotherapy, prophylactic irradiation and intrathecal therapy in the treatment of this disease. Complete remission rates by using these combination chemotherapies have risen to 85–90% and two year survival rates have risen to 70–76% (Glatstein *et al.*, 1974; Link *et al.*, 1985; Sullivan *et al.*, 1985).

This paper reports the efficacy of combined chemotherapy 'modified and less aggressive LSA2-L2 regimen' and the degree to which the results were influenced by the extent and histopathological subtypes of the disease.

### Patients and methods

Between January 1983 and December 1986, 76 children with non-Hodgkin's lymphoma (except those diagnosed as having Burkitt's lymphoma by the institutional pathologist) were treated at Hacettepe Children's Hospital Oncology Unit. All patients were less than 18 years of age. There were 49 males and 27 females. Male/female ratio was 1.8 and median age was 6 years.

The extent of disease was determined (by history, physical examination, blood count, bone marrow aspiration, spinal-fluid cell count and smear for white-cell morphology) two dimensional chest X-rays, bone scan of skeletal survey, intravenous pyelogram, and in some patients abdominal ultrasonography and computerized axial tomography. Routine lymphangiogram and staging laparotomy were not performed, although abdominal patients underwent a laparotomy for diagnostic, therapeutic or palliative reasons.

The extent of disease at diagnosis was categorized according to the St. Jude staging system, in which localised disease (stage I and II) was defined as a tumour limited anatomically either to a single extranodal site, with or without positive regional nodes, or to lymph nodes in one or two adjacent lymphatic regions. Grossly complete excision was required for tumours arising in the gastrointestinal tract to be classified as localised disease. All other tumours were classified as non-localized (stage III and IV) disease. Included in this category was any patient with a mediastinal mass a diagnosis.

More than half of the patients had abdominal disease (53%) either nodal or gastrointestinal primary lesions (Table III). Thirty-two percent of the patients had mediastinal or widespread disease and 19% had involvement of the bone marrow or central nervous system at diagnosis, in addition to disseminated disease at other sites (stage IV) (Table IV). Seventy-nine percent of the patients were non-localized (stage III and IV) and 21% were localized (stage I and II) at the time of diagnosis according to the Murphy's classification.

### *Histologic classification of tumour type*

Tissue suitable for histologic examination was obtained from all patients. Cytologic slides were reviewed by the institutional pathologist. Most of the unclassified non-Hodgkin's lymphoma slides came from some other hospitals. Therefore our pathologists could not specify subgroups of the disease. The lymphomas were classified according to the Rappaport system. On the basis of pattern (nodular or diffuse) and cytomorphology histologic material was classified as one of four types; lymphoblastic, histiocytic, undifferentiated non-Burkitt's and undifferentiated Burkitt's type. In this study undifferentiated Burkitt's patients were considered ineligible.

### *Surgery*

Grossly complete tumour excision was attempted in patients with apparently localized (stage I and II) disease. A biopsy was generally performed in patients with non-localized disease. In children with apparently localized disease arising in the gastrointestinal tract, exploratory surgery was performed and grossly complete excision was undertaken whenever possible. In patients who had widespread disease in the abdomen, but not elsewhere at diagnosis, a laparotomy and biopsy were performed, occasionally with a debulking procedure. In patients with large mediastinal mass and pleural effusion and no accessible extrathoracic disease a

Correspondence: M. Büyükpamukçu.
Received 21 October 1986; and in revised form, 22 June 1987.

cytologic diagnosis was made on the basis of examination of the pleural fluid.

*Chemotherapy*

Eligible patients were assigned according to their clinical stage. Stage I and II patients were treated with Regimen I, and stage III and IV patients with Regimen II. Regimen I consisted of an induction phase followed by maintenance (Table I). Regimen II had three phases; induction, consolidation and cycles of maintenance (Table II). Central nervous system prophylaxis in regimen II consisted of repeated injections of intrathecal methotrexate, cytosine arabinoside and hydrocortisone. Prophylactic CNS irradiation was only given to the intrathoracic lymphoma patients. Both regimens were followed for 24 months from the first day of induction.

*Statistical analysis*

Analysis of the response to treatment focused on the time from entry into the study to the first adverse event, if any. Events defined as adverse were: no response by the completion of induction therapy, a relapse of any kind, and death. Any adverse event was considered a treatment failure. The period from entry into the study to the first adverse event is referred to below as failure-free survival. The product-limit method was used to estimate the distribution of failure-free survival and of overall survival (Peto *et al.*, 1977).

### Results

Complete remission rate was 86.8% at the end of induction therapy. Survival rate was 80.1% in patients who had complete remission at the end of induction therapy and two year continuous complete remission rate was 64.3% in those patients. Failure-free survival for all patients was estimated to be 59.2% at 24 months and overall survival was 72.3% at 24 months (Figure 1). Length of follow up was 17–52 months. At the time of writing, the median follow-up for patients who have had no adverse event is 28 months.

The extent of the disease at diagnosis was an important indicator of treatment response in our study. Among the patients with stage I and II the failure-free survival rate was 81.2% while it was 53.3% in stage III and IV patients at 24 months (Figure 2). Failure-free survival was 50% in patients with poorly differentiated lymphoma, 67.8% in patients with undifferentiated non-Burkitt's group and 75% in the unclassified group (Figure 3).

Failure-free survival rate was 53.8% in patients with mediastinal disease and 68.3% in those with abdominal disease at 24 months (not statistically significantly different) but was only 20% in widespread non-Hodgkin's lymphoma at diagnosis (Figure 4). Thus, failure-free survival is clearly related to the primary site of the disease in this series. The relationship of primary site to relapse rate is shown in Table VI. The relapse rate was 46.1% at 20 months in patients with mediastinal non-Hodgkin's lymphoma and 24.3% in abdominal and 60% in widespread cases.

**Table I** Chemotherapy regimen for stage I and II NHL

| | |
|---|---|
| INDUCTION: | |
| Cyclophosphamide | 1200 mg m$^{-2}$ i.v., days 1, 21 and 42 |
| Oncovin (vincristine) | 1.5 mg m$^{-2}$ (max. 2 mg) i.v., days 1, 7, 14, 21, 28 and 35 |
| Prednisone | 40 mg m$^{-2}$ per day, p.o. divided 4, 6 hourly doses. on days 1–28 decreasing to 0 on days 29–34 |
| MAINTENANCE (For 2 years): | |
| Methotrexate | 30 mg m$^{-2}$ p.o. weekly |
| 6 MP (Purinethol) | 75 mg m$^{-2}$ p.o. daily |

**Table II** Chemotherapy regimen for stage III and IV NHL

| | |
|---|---|
| INDUCTION: | |
| Cyclophosphamide | 1200 mg m$^{-2}$ i.v., days 1, 21 and 42 |
| Oncovin (vincristine) | 1.5 mg m$^{-2}$ (max. 2 mg) i.v., days, 1, 7, 14, 21, 28 and 35 |
| Adriamycin | 45 mg m$^{-2}$ i.v., days 1, 21 and 42 |
| Prednisone | 40 mg m$^{-2}$ per day, p.o., divided 4, 6 hourly doses on days 1–28 decreasing to 0 on days 29–34 |
| Cytosine arabinoside | 60 mg m$^{-2}$ intrathecally, days 1, 7, 14, 21 and 28 |
| Hydrocortisone | 30 mg m$^{-2}$ intrathecally, days 1, 7, 14, 21 and 28 |
| Methotrexate | 15 mg m$^{-2}$ (max. 15 mg) intrathecally, days 1, 7, 14, 21 and 28 |
| CONSOLIDATION: | |
| Cytosine arabinoside | 100 mg m$^{-2}$ i.v., days 1–5 (mon.–fri.) for 2 weeks |
| L-Asparaginase | 6000 U m$^{-2}$ i.v., daily for 10 days between two cytosine arabinoside cycles |
| Radiotherapy | 24 Gy cranial for the patients with thoracic involvement (especially T-cell) |
| MAINTENANCE CYCLES (For two years): | |
| Cyclophosphamide | 1200 mg m$^{-2}$ i.v. day 1 |
| Oncovin (vincristine) | 1.5 mg m$^{-2}$ (max. 2 mg) i.v., day 1 |
| Prednisone | 40 mg m$^{-2}$ p.o., days 1–5 with doses decreasing to 0 on days 6–8 |
| Cytosine arabinoside | 60 mg m$^{-2}$ intrathecally, day 1 |
| Hydrocortisone | 30 mg m$^{-2}$ intrathecally, day 1 |
| Methotrexate | 15 mg m$^{-2}$ (max. 15 mg), intrathecally, day 1 |
| (Repeat maintenance cycles every 2 months) | |
| Methotrexate | 30 mg m$^{-2}$ p.o. weekly |
| 6MP (Purinethol) | 75 mg m$^{-2}$ p.o. daily |
| (except on weeks that patients are taking the maintenance cycles) | |

**Table III** Primary sites of presentation in patients with NHL

| *Site of presentation* | *Number of patients* (%) |
|---|---|
| Peripheral | 3 (4) |
| Mediastinal | 13 (17) |
| Abdominal | 41 (54) |
| Widespread | 12 (16) |
| Others | 7 (9) |
| Total | 76 (100) |

**Table IV** The clinical stages of patients with non-Hodgkin's lymphoma

| *Stage* | *No of patients* (%) |
|---|---|
| I | 6 (8) |
| II | 10 (13) |
| III | 45 (59) |
| IV | 15 (20) |

**Table V** Pathological classification of non-Hodgkin's lymphoma patients (Modified Rappaport Classification)

| *Histopathologic subtypes* | *No. of patients* | *Percent* |
|---|---|---|
| Diffuse poorly differentiated lymphocytic (DPDL)[a] | 42 | 55 |
| Diffuse histiocytic (DH) | 2 | 3 |
| Diffuse undifferentiated non-Burkitt (DUNB) | 16 | 21 |
| Diffuse unclassified (Unclass.) | 16 | 21 |
| Total | 76 | 100 |

[a]Including 11 lymphoblastic lymphoma.

**Table VI** Relationship of primary site to relapse rates in patients with non-Hodgkin's lymphoma

| *Primary site* | *No. of patients* | *Patients relapsed* | |
|---|---|---|---|
| | | *No.* | *Percent* |
| Peripheral | 3 | 1 | — |
| Mediastinal | 13 | 6 | 46.1 |
| Abdominal | 37 | 9 | 24.3 |
| Widespread | 10 | 6 | 60.0 |
| Others | 7 | 3 | 42.0 |
| Total | 70 | 25 | 35.7 |

**Figure 1** Overall and failure-free survival in 76 patients with childhood non-Hodgkin's lymphoma.

**Figure 2** Overall and failure-free survival according to extent of disease at diagnosis.

**Figure 3** Failure-free survival according to the histopathological subgroups.

**Figure 4** Failure-free survival according to primary sites.

### *Toxicity*

Eleven patients died during treatment as a result of treatment-related sepsis, four early in the induction phase, one with systemic varicella in remission, one with iatrogenic purulent meningitis in remission and the remaining 5 patients during relapse with systemic bacterial and viral infections. Haematologic toxicities were neutropenia (28.9%) and thrombocytopenia (4%). After these deaths, we gave prophylactic antibiotic therapy (either trimethoprim-sulfamethoxozole or metragyl) during induction to all stage III and IV patients.

### Discussion

Non-Hodgkin's lymphomas in childhood differ considerably from those in adults in that nodular patterns are rare in children. Non-Hodgkin's lymphomas grow rapidly and disseminate early in children and the majority of patients have widespread disease at diagnosis. In the past, despite therapy, most children had a relapse within a year of diagnosis and long term survival was only about 15%.

The prognosis for childhood non-Hodgkin's lymphoma has improved dramatically in the last two decades. Because of similarities between non-Hodgkin's lymphoma and acute lymphoblastic leukaemia, a number of investigators adopted a more aggressive approach using anti-leukaemic chemotherapy in addition to radiotherapy in an attempt to reduce the incidence of distant metastasis and to prolong remission duration. This approach resulted in prolonged remission in children with localized non-Hodgkin's lymphoma and reduced the incidence of leukaemic transformation (Glatstein *et al.*, 1974; Meadows *et al.*, 1980, Büyükpamukçu *et al.*, 1983).

The addition of new chemotherapeutic agents and the application of novel combinations of drugs have led to gratifying improvements in survival for children with advanced non-Hodgkin's lymphoma too. Wollner *et al.* (1976; 1979) treated 39 children with a multidrug leukaemia programme intensified with cyclophosphamide and radiation treatment (LSA2-L2). They reported a long term disease-free survival rate of 73%. Using a leukaemia regimen intensified with cyclophosphamide and doxorubicin for non-localized advanced disease Murphy and Hustu (1980) obtained a disease free survival rate of 90% in patients with localized disease and 39% in those with disseminated disease, with 2-year relapse-free survival of 55%. In the CCSG pilot study with COMP Meadows *et al.* (1980) obtained a two year survival rate of 68%, without prohibitive toxicity. Anderson *et al.* (1983) compared modified LSA2-L2 and COMP regimens and found results for localized patients did not differ according to the regimen used, but patients treated with COMP had less toxicity. Significant treatment differences were observed in patients with non-localized disease but only within the lymphoblastic subgroups. Modified LSA2-L2 was more effective in lymphoblastic lymphoma, whereas COMP was more effective in the non-lymphoblastic groups (undifferentiated Burkitt, non-Burkitt, and histiocytic).

Mott *et al.* (1984*a*) obtained 66% failure-free survival with combination chemotherapy and low dose radiotherapy in T-cell lymphoma. They also reported 75% failure-free survival for localized disease (stage I and II), and 51% for generalized disease (stage III and IV) at four years (Mott *et al.*, 1984*b*). In the latter group (with no mediastinal primary), there was no benefit to patients randomized at the end of induction chemotherapy to receive adjuvant low dose radiation to sites of previous bulky disease when compared to those not receiving such radiation.

In our study, the extent of the disease at diagnosis was an important determinant of the overall response to therapy. Among the patients with localized disease, the 2 year failure-free survival was 82.1%, while it was 53.3% in those with disseminated disease.

There was also a relationship between the primary site of the tumour and the failure-free survival rate. Among the patients with mediastinal disease, 2 year survival rate was 53.8% and while in abdominal disease, it was 73.2%.

Our diffuse poorly differentiated lymphoblastic lymphoma group's failure-free survival rate (50% in 2-year) was lower than that of the undifferentiated non-Burkitt group (66.8%) but the difference was not statistically significant.

The overall results of this trial represent a significant improvement over our own institution's historical controls. We reported 31% 2-year survival in patients treated from 1972–1978 (Büyükpamukçu *et al.*, 1983) while our estimate of the proportion of all patients entered in the present study surviving at two years is 72.4%.

Despite this appreciable progress, it is nevertheless clear that treatment for certain subgroups (the poorly differentiated lymphocytic lymphoma and primary mediastinal form) remains unsatisfactory. For these we need more intensive or better treatment.

### References

ANDERSON, J.R., WILSON, J.F., JENKIN, D.T. & 8 others (1983). Childhood non-Hodgkin's lymphoma. The results of a randomized therapeutic trial comparing a 4-drug regimen (COMP) with a 10-drug regimen ($LSA_2$-$L_2$). *N. Engl. J. Med.*, **308**, 559.

BÜYÜKPAMUKÇU, M., BÜYÜKGEBIZ, A., TEKINALP, G., BÜYÜKPAMUKÇU, N. & ÇEVIK, N. (1983). The results of combination chemotherapies in childhood non-Hodgkin's lymphoma. *Kanser*, **13**, 31.

GLATSTEIN, E., KIM, H., DONALDSON, S.S. & 5 others (1974). Non-Hodgkin's lymphomas IV. Results of treatment in childhood. *Cancer*, **34**, 204.

LINK, M.P. (1985). Non-Hodgkin's lymphoma in children. *Pediatr. Clin. North Am.*, **32**, 699.

MEADOWS, A.T., JENKIN, R.D., ANDERSON, J. & 8 others (1980). A new therapy schedule for pediatric non-Hodgkin's lymphoma. Toxicity and preliminary results. *Med. Pediatr. Oncol.*, **8**, 15.

MOTT, M.G., CHESSELLS, J.M., WILLOUGHBY, M.N.L. & 5 others (1984*a*). Adjuvant low dose radiation in childhood T-cell leukaemia/lymphoma. *Br. J. Cancer*, **50**, 457.

MOTT, M.G., EDEN, D.B. & PALMER, M.K. (1984*b*). Adjuvant low dose radiation in childhood non-Hodgkin's lymphoma. *Br. J. Cancer*, **50**, 463.

MURPY, S.B. & HUSTU, H.O. (1980). A randomized trial of combined modality therapy of childhood non-Hodgkin's lymphoma. *Cancer*, **45**, 630.

PETO, R., PIKE, M.C., ARMITAGE, P. & 7 others (1977). Design and analysis of randomized clinical trials requiring prolonged observation of each patient. II. Analysis and examples. *Br. J. Cancer*, **35**, 1.

SULLIVAN, M.P., BOYETT, J., PULLEN, J. & 6 others (1985). Pediatric Oncology Group Experience with modified $LSA_2$-$L_2$ therapy in 107 children with non-Hodgkin's lymphoma (Burkitt's lymphoma excluded). *Cancer*, **55**, 323.

WOLLNER, N., BURCHENAL, J.H., LIEBERMAN, P.H., EXELBY, D., D'ANGIO, G. & MURPY, M.L. (1976). Non-Hodgkin's lymphoma in children. A comparative study of two modalities of therapy. *Cancer*, **37**, 123.

WOLLNER, N., EXELBY, P.R. & LIEBERMAN, P.H. (1979). Non-Hodgkin's lymphoma in children. A progress report on the original patients treated with the $LSA_2$-$L_2$ protocol. *Cancer*, **44**, 1990.